# Guiding syringe selection for intravitreal injections: injectability and stability analysis of compounded pegcetacoplan (SYFOVRE) and the broader implications for high-viscosity ophthalmic therapies

**DOI:** 10.1186/s40942-026-00832-3

**Published:** 2026-03-12

**Authors:** Tina Felfeli, Natalie M. Lane, Efrem D. Mandelcorn

**Affiliations:** 1 Department of Ophthalmology and Vision Sciences, University of Toronto, Toronto, ON Canada; 2 Department of Medicine, Royal College of Surgeons in Ireland, Dublin, Leinster, Ireland; 3 Toronto Western Hospital, University Health Network, Toronto, ON Canada

**Keywords:** SYFOVRE, Pegcetacoplan, Faricimab, Aflibercept, Syringe selection, Age-related macular degeneration, Anti-VEGF, Silicone oil, High viscosity formulations, Injectability

## Abstract

**Purpose:**

SYFOVRE, the brand name of pegcetacoplan and the first FDA-approved treatment for geographic atrophy arising from dry age-related macular degeneration, is currently available only in vial format. Consequently, it is often compounded and transferred into syringes. This study aims to identify the optimal syringe type for both storage and intravitreal injection (IVI) of medium- and high-viscosity formulations such as SYFOVRE.

**Methods:**

SYFOVRE was prepared in four syringe types and stored under refrigeration in amber bags for up to 110 days. Samples were evaluated for potency, aggregation, protein integrity, binding activity, pH, appearance, sterility, and container closure integrity. Syringe particulate contribution was examined using light obscuration. Injectability of each syringe was assessed through administration of 15 cP and 120 cP viscosity mimics into the vitreous of human cadaver eyes, and injection force was recorded over time.

**Results:**

Pegcetacoplan remained stable across all syringe types for 110 days. The ClearJect (0.5 mL) syringe exceeded particulate safety limits as defined by USP <789>. Ophthalmic-specific syringes (Zero Residual [0.2 mL] and StaClear [0.25 mL]) required significantly lower injection forces than the BD (1 mL) and ClearJect (0.5 mL) (*p* < 0.0001).

**Conclusion:**

While all syringes preserved stability of SYFOVRE, injection force increased with higher viscosity. The borosilicate syringe (ClearJect) failed particulate testing, disqualifying it as a viable IVI option. Low-volume ophthalmic-specific syringes (Zero Residual and StaClear) demonstrated superior performance, suggesting they should be used for the safer delivery of intravitreal therapies. Notably, Zero Residual is the only IVI-indicated syringe that eliminates silicone oil–associated risks.

**Supplementary Information:**

The online version contains supplementary material available at 10.1186/s40942-026-00832-3.

## Introduction

In 2016, approximately 7 million intravitreal injections (IVIs) were administered in the United States alone [1], primarily for vision-saving anti-VEGF therapies. Since these retinal disorders are more prevalent in older populations, the global shift toward an aging population is expected to drive a continued increase in the number of IVIs. In the context of rising IVI volumes, syringe choice is an often underrecognized variable with meaningful implications for treatment efficacy and patient safety. Advances in polymer prefilled syringe technologies, like SiO-free propylene, cyclic olefin polymer and cyclic olefin copolymer, have been introduced as promising alternatives to traditional silicone oil-lubricated borosilicate syringes for biologic drug delivery. These polymer-based systems offer several advantages over conventional glass syringes, including significantly reduced protein adsorption due to their non-polar, hydrophobic surfaces [23], some eliminate the use of silicone oil (reviewed in Melo et al., 2021), lower extractables and leachables [24], and reduced formation of protein aggregates compared to glass syringes [25]. As emphasized by Melo et al. (2021), the selection of appropriate materials for syringes and needles is critical for ensuring optimal drug delivery and minimizing potential complications in intravitreal and other parenteral administration routes. However, ophthalmologists have traditionally used general-purpose 1 mL syringes not optimized for IVI, either filling the syringes via vial-to-syringe transfer or, more commonly, using syringes filled by compounding pharmacies to ensure sterility and optimize clinical workflow. Syringes filled by compounding pharmacies are then assigned a beyond use date based on either local regulatory maximums or based on proprietary evidence-based evaluation. While some manufacturers have introduced prefilled syringes (PFSs) for various medications, many products remain available only in vial format, including the pegcetacoplan intravitreal injection (SYFOVRE), which is supplied as a vial rather than a prefilled syringe. Additionally, the treatment of retinal diseases often requires repeated IVIs, which increases the risk of syringe-related adverse events such as sterile inflammation [2], intraocular pressure (IOP) spikes [3] and silicone oil (SiO) floaters (occasionally requiring vitrectomy) [4–7]. A significant number of these events have been traced to SiO residues in syringe components [2, 4, 6–11] and recently the use of siliconized needles in IVIs was linked to SiO floaters in human eyes [12] highlighting the need for a completely SiO-free injection system. The combination of syringe-associated adverse events, lack of manufacturer validated PFSs, and the need for compounding pharmacies to select syringes that support preparation stability to extend beyond use dates underscores the urgent need for comprehensive syringe comparisons.

In 2021, dry AMD affected an estimated 36 million people globally and plays a key role in macular degeneration, which is the leading cause of blindness in high income countries [13]. In its advanced stages, dry AMD can progress to geographic atrophy (GA), a severe form of retinal degeneration characterized by inflammation-mediated cell death of photoreceptors, retinal pigment epithelium, and the choriocapillaris, leading to atrophic lesions. GA is associated with irreversible vision loss and blindness, profoundly impacting patients’ overall quality of life [14]. Anti-VEGF treatments such as Eylea, Avastin and Vabysmo, have been available since 2004 for neovascular (wet) AMD. However, it was not until 2023 that SYFOVRE became the first FDA-approved treatment for GA arising from dry AMD. The active ingredient in SYFOVRE, pegcetacoplan, is a PEGylated bicyclic peptide that selectively binds complement component C3 and its activation fragment C3b, attenuating complement-driven inflammation and slowing the progression of GA.

Concentrated therapies, like SYFOVRE (15 mg/0.10 mL), are designed to maximize drug delivery within the constrained IVI volume range (50–100 µL) [15], supporting extended dosing intervals and reducing treatment burden. However, increasing concentrations of biotherapeutics leads to exponentially higher viscosities [16], amplifying differences in syringe injectability – the broad set of characteristics that contribute to ease a formulation can be administered using a given medical device – and underscoring the need for careful syringe selection to ensure efficient and reproducible delivery of therapeutics.

Recently the rheological properties of higher viscosity formulations have been implicated in introducing SiO floaters during injection [17–20]. This began with early reports that observed SiO floaters in the eyes of patients that received intravitreal pegcetacoplan [17, 18]. In a case series by Dessouki et al. (2023), 29% (16/55) of patients receiving intravitreal pegcetacoplan using the siliconized 1 mL McKesson Luer-lock syringe reported floaters, which were subsequently identified as presumed silicone oil droplets. Interestingly, over the same time period 0% (0/512) of patients at the same clinic reported floaters when receiving intravitreal injections of farcimab using the same syringe system [18]. Several groups have speculated that the combination of increased formulation viscosity and greater manual agitation to remove air bubbles likely promotes SiO droplet release and dispersion in pegcetacoplan formulations [17–19]. Higher viscosity formulations (faricimab, pegcetacoplan, and avacincapted pegol) have also been observed to entrain more silicone oil from siliconized syringes than ultrapure water [20]. As protein therapeutics continue to trend toward higher viscosities, these observations underscore the growing importance of using low-silicone or silicone oil–free syringe systems to minimize particulate contamination and associated visual disturbances.

**Fig. 1:**
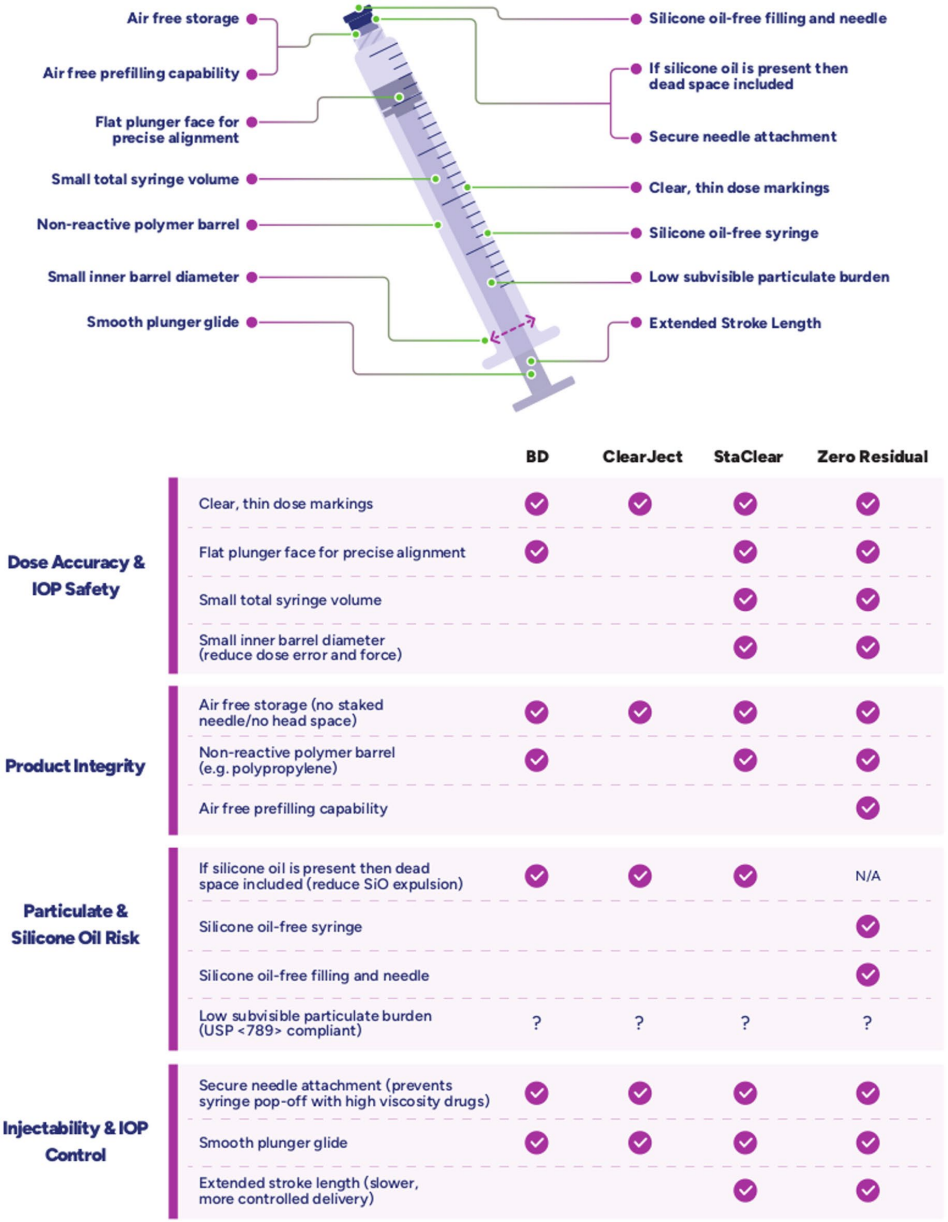
Ideal syringe features for intravitreal syringes and presence of those features in the syringes used in this study

Syringes selected for intravitreal injection should be designed to prioritize patient safety through dose accuracy, maintenance of drug product integrity and low particulate shedding while providing consistent and low-force injectability (Fig. 1).

Dose accuracy is a critical factor in minimizing overdose-related IOP elevations. Repeated IOP spikes have been associated with thinning of the retinal nerve fiber layer [3] and an increased risk of glaucoma [21, 22]. The risk of overdosing can be reduced through clear, fine dose graduations, a flat plunger face to enable accurate user alignment with volume markings, small total syringe volume and a smaller barrel inner diameter, as minor plunger misalignments in larger-diameter barrels translate into proportionally larger dosing errors [23].

Drug product integrity could be affected by syringe material incompatibility or exposure to air-liquid interfaces. For example, tungsten that leached from borosilicate syringes has been observed to contribute to elevated epoetin alfa protein aggregation [24]. Air-liquid interface in syringes with staked-on needles has been shown to promote protein aggregation [25, 26] and thereby reduce product integrity. Thus, careful material selection as well as air-free prefilling and storage helps prevent protein aggregation and support product stability.

Particulate-related adverse events, including silicone oil (SiO) release, have been associated with inflammatory responses [9–11] and symptomatic SiO floaters [5–7, 27]. To mitigate the risk of particulate release during IVIs, the syringes used for IVIs should be manufactured from stable materials that contribute few particulates to drug products and avoid the use of SiO as a lubricant for the plunger.

Finally, smooth and reliable injection performance is essential for clinical usability across both low- and high-viscosity formulations. Injection reliability is enhanced by a secure needle attachment, which minimizes the risk of needle pop-off, an issue that becomes increasingly critical at the higher injection force required for viscous formulations. In addition, a longer stroke length with a smaller barrel diameter enables a slower, more controlled plunger advancement with a lower force required for dose delivery.

While extensive research has evaluated the stability of antibody-based protein therapeutics in various syringe types [28–30], little research has been performed on the stability of PEGylated proteins, like pegcetacoplan. Given the increasing clinical use of pegcetacoplan and its structural distinction from previously studied biologics, there is a need to evaluate how its unique design may impact stability in compounded syringes.

This convergence of emerging high-viscosity therapeutics and syringe technologies represents a shift in IVI administration. This study aimed to identify syringes that offer optimal injectability across a range of viscosities while maintaining low particulate shedding. SYFOVRE was used as a model for PEGylated and high viscosity peptide therapies to investigate the impact of syringe type on post-compounding stability and bioactivity. Syringes chosen for analysis included two small volume syringes indicated for IVIs, StaClear (0.25 mL) and Zero Residual (0.2 mL), as well as a traditional BD syringe (1 mL) and a ClearJect borosilicate syringe (0.5 mL). These findings are intended to guide syringe selection in clinical settings and support the safe IVI delivery of high-viscosity therapies for retinal disease.

## Materials and methods

### Compounding

Pegcetacoplan 150 mg/mL (SYFOVRE™, Apellis) was aseptically compounded under NAPRA-compliant conditions, into four syringe types commonly used for intravitreal injection: 1 mL BD Luer-Lock™ SiO lubricated (BD, NJ, USA), 0.2 mL Zero Residual SiO-free (SJJ Solutions, The Hague, Netherlands), 0.25 mL StaClear immobilized SiO (StaClear, NC, USA), and 0.5 mL ClearJect baked on SiO (LUVO Medical Technologies, Inc, ON, CAN). Pegcetacoplan was drawn directly from the manufactured vial into BD, StaClear, and ClearJect syringes using BD Blunt Fill Needles and filled to a volume of 0.12 mL. For the Zero Residual syringes, pegcetacoplan was first drawn into a silicone oil-free Bubble Adaptor (SJJ, Netherlands) and then air-free prefilled into the syringes. All BD, StaClear, and ClearJect syringes were capped with Sol-M tip caps (Sol-M, IL, USA) while Zero Residual syringes used SiO-free tip caps (SJJ, Netherlands). Following compounding in ISO 5 laminar flow hoods, syringes were visually inspected, individually sealed in labeled amber bags under ISO 7 conditions, and stored at 2–8 °C until analysis.

For the injection force experiment, syringes were compounded using identical techniques to that above; but a 15 cP or 120 cP viscosity mimic was used instead of a biotherapeutic.

### Preparation of viscosity mimic

The theoretical viscosities of the mimic solutions were calculated using the Arrhenius equation to determine the relative volumes of each reagent in the final mixture. The SYFOVRE viscosity mimic was prepared using a combination of polyethylene glycol 400 (PEG-400) (Medisca) and polyethylene glycol 600 (PEG-600) (Sigma Aldrich). PEG-400 and PEG-600 were mixed at a ratio of 4:1 to create a 120 cP mimic solution. The mid-range viscosity mimic was prepared using PEG-400, which was mixed with water at a ratio of 11:9 to create the 15 cP mimic solution. The viscosity of both mixtures were confirmed using a Cannon-Manning Semi-Micro Viscometer.

### Analytical methods

Analytical materials and methods used in this study are described in full in supplementary file 1.

## Results

### Particulate matter in syringes compounded with sterile water for injection

**Fig. 2:**
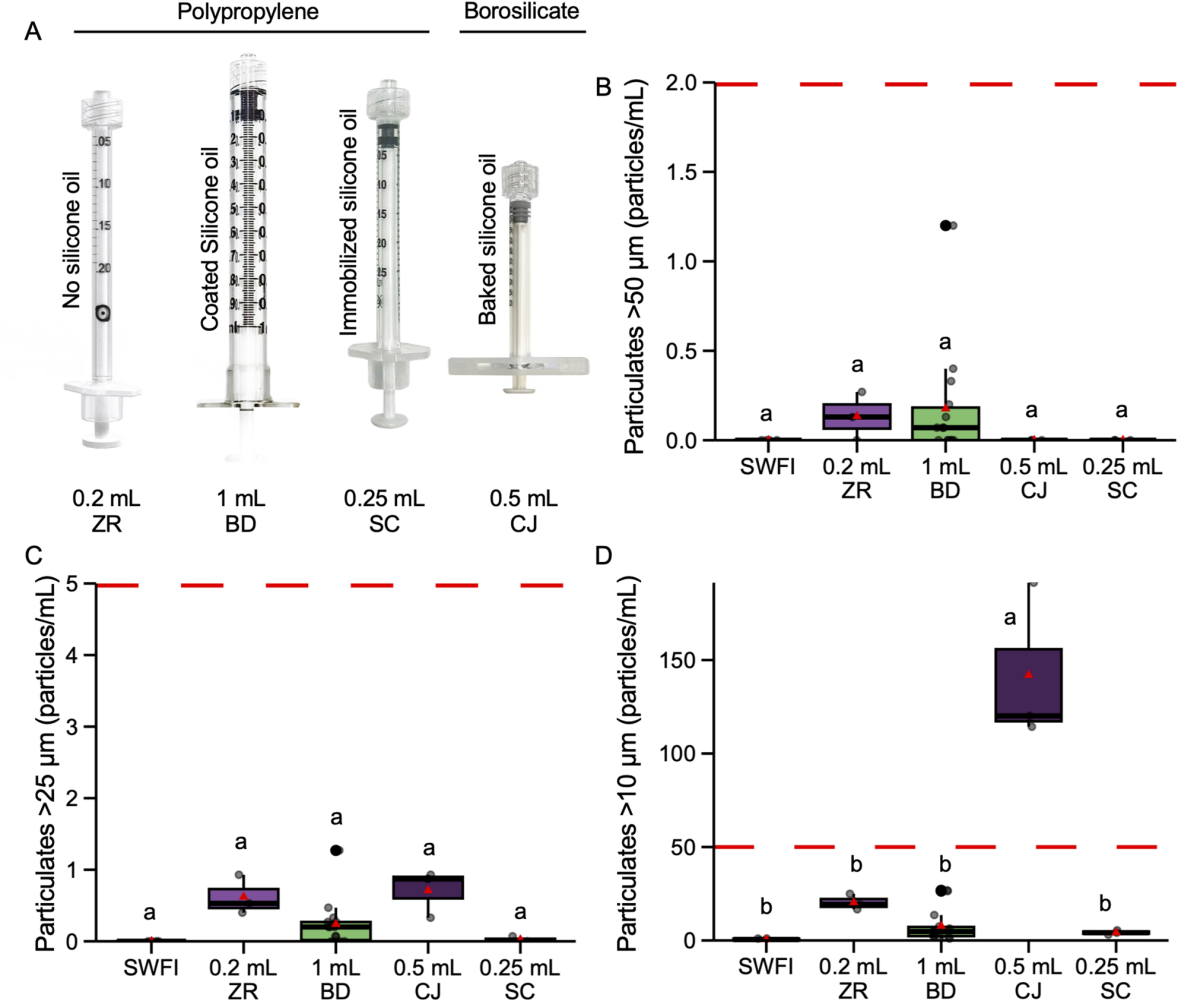
Particulate matter testing of syringes compounded with sterile water for injection. (**A**) An image showing each of the syringes used in this study. From left to right, 0.2 mL Zero Residual (ZR), 1 mL BD Luer-Lock (BD), 0.25 mL StaClear (SC), and 0.5 mL ClearJect (CJ). (**B-D**) For each replicate, 234 syringes were compounded with 0.15 mL sterile water for injection and then pooled and subjected to USP < 789>-compliant particulate matter testing. The number and size of particulates was measured and categorized based on the diameter of the observed particle. The number of particles greater than 50 μm (**B**), 25 μm (**C**), and 10 μm (**D**) were counted for each syringe type and uncompounded sterile water for injection. The data is shown in a boxplot, where the limits of each box indicate the 25th and 75th percentiles, median is indicated by a horizontal line, a red triangle depicts the mean, and data points are shown as transparent black dots. A red dotted line depicts the particulate matter cut-off for each size category based on USP < 789 > acceptance criteria. Different letters indicate statistically significant differences (ANOVA, Tukey’s HSD, p-value < 0.05)

USP < 789>, Particulate Matter in Ophthalmic Solutions, establishes strict limits on both the size and concentration of particulates permitted in ophthalmic formulations to minimize the risk of adverse patient outcomes. Particulates can originate from the syringe (e.g., silicone oil, plastic or stopper shedding) or the formulation itself (e.g., protein aggregation, excipient crystallization). Ophthalmic-specific syringes should therefore contribute minimally to overall particulate levels. To assess syringe-derived particulates, syringes of each type (Fig. 2A) were prefilled with 0.15 mL of sterile water for injection, a volume selected to reflect a realistic compounded syringe volume for SYFOVRE, accounting for the labeled 0.10 mL intravitreal injection dose and necessary syringe overfill. Each experimental replicate consisted of 234 syringes, which were pooled to achieve the 35 mL required to perform particulate analysis of each experimental replicate in technical triplicate. Particulate analysis was evaluated according to USP < 789 > guidelines. Specifically, the standard permits higher concentrations of smaller particles (≤ 50 particles/mL ≥ 10 μm) and increasingly stringent limits for larger particles (≤ 5 particles/mL ≥ 25 μm and ≤ 2 particles/mL ≥ 50 μm). In the largest particle size category (particles greater than 50 μm in diameter), all syringes had < 0.25 particles/mL and fell within USP < 789 > guidelines (Fig. 2B). Similarly, for particles greater than 25 μm in diameter, all syringes were within specifications and had < 1 particles/mL (Fig. 2C). In the smallest particle size category, particles greater than 10 μm in diameter, the borosilicate ClearJect syringe had ~ 120 particles/mL, more than twice the USP < 789 > limit. These results implicate the ClearJect syringe as contributing particulate matter to contained solutions beyond USP safety thresholds, suggesting that this syringe would negatively impact the quality of ophthalmic preparations. In contrast, the Zero Residual and BD syringes fell within USP < 789 > specifications and had comparable particulate concentrations (Fig. 2D). While the StaClear syringe met USP < 789 > acceptance criteria, visible residue deposits were observed on the inner barrel following plunger depression (Figure S1). Future studies should examine and investigate the nature of this residue.

### Syringe injectability assessment with human cadaver eyes

**Fig. 3:**
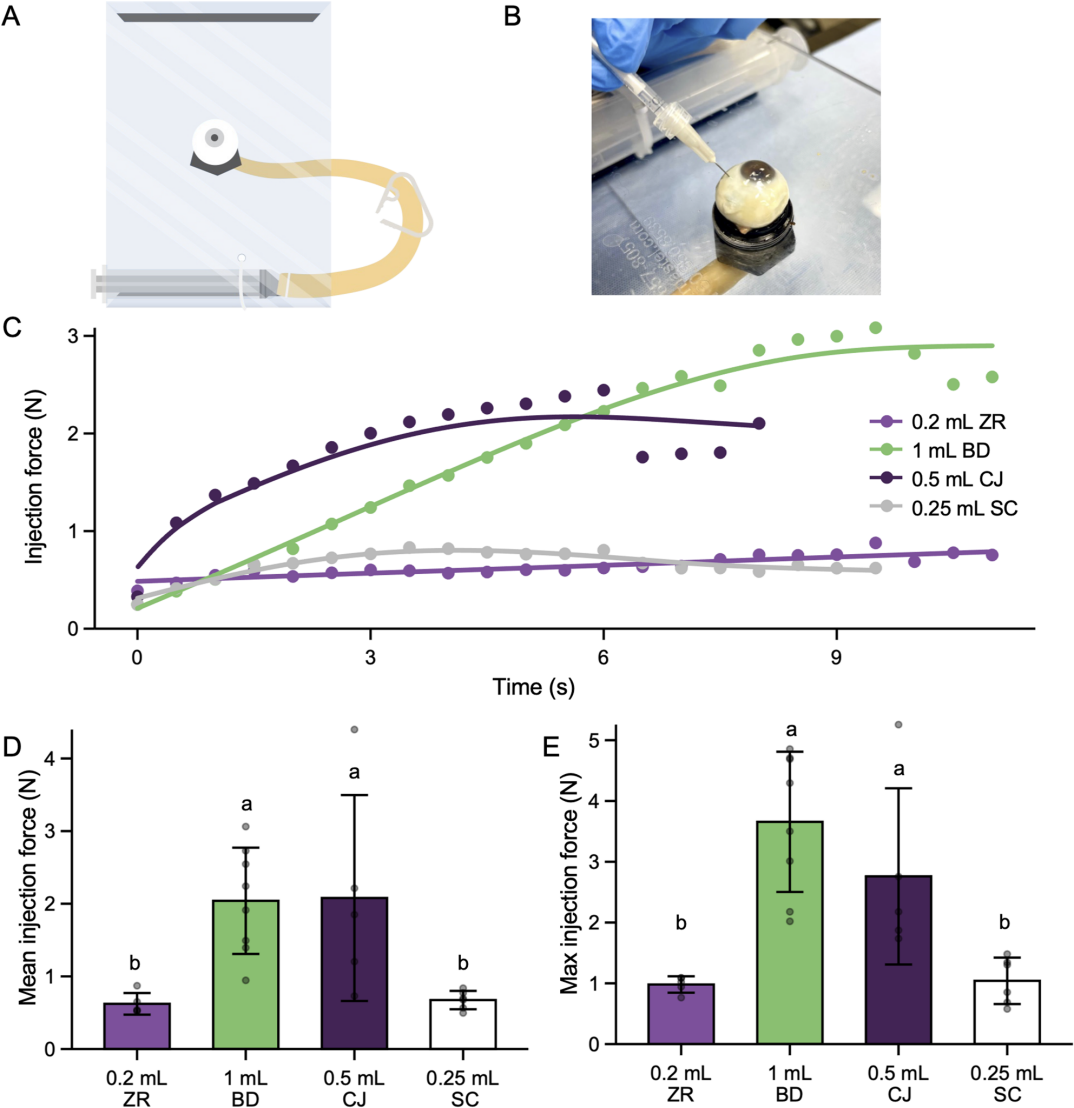
Injection force of various syringes for 120 cP solution delivered into human cadaver eye vitreous. 0.2 mL Zero Residual (ZR), 1 mL BD Luer-Lock (BD), 0.25 mL StaClear (SC), and 0.5 mL ClearJect (CJ) syringes were compounded with either 0.12 mL of a 15 cP (supplementary) or 120 cP (above) viscosity mimic solution. A BD 27G x ½” precision glide needle was affixed, and the device was primed to 0.1 mL prior to injection into the vitreous of human cadaver eyes. All injections were performed by the same ophthalmologist. (**A**) Apparatus for the artificial induction of physiologically relevant IOP in the ocular globe. (**B**) Demonstration of the intravitreal injection with the 0.2 mL ZR syringe. (**C**) Injection force over time of various syringes filled with 120 cP SYFOVRE viscosity mimic solution. Dots represent the mean injection force at 0.5 s intervals. The lines represent a non-linear regression fit for injection force over time for each syringe type. The mean (**D**) and max (**E**) injection force for intravitreal injections. Bars represent the aggregate average of the mean and max injection forces for multiple injections and error bars indicate the standard deviation. Dots represent the mean injection force for individual injections. Different letters indicate statistically significant differences (ANOVA, Tukey’s HSD, p-value < 0.05)

Older formulations like Avastin and Eylea have a viscosity of approximately 1 cP (Table S1) at room temperature, similar to water, whereas newer formulations like Vabysmo and SYFOVRE exhibit significantly higher viscosities of 9 and 120 cP, respectively (Table S1). As this study aims to examine the injectability of the new higher viscosity solutions, a 15 cP mid-range viscosity mimic solution and a 120 cP SYFOVRE viscosity mimic solution were prepared. For these viscosity mimics, polyethylene glycol (PEG-400 and PEG-600) was selected as a thickening agent to mimic the PEGylated portion of SYFOVRE. Each syringe type was pre-filled with either 15 cP (mid-range viscosity mimic) or 120 cP (SYFOVRE mimic) solutions and a trained ophthalmologist delivered the solution into the vitreous of human cadaver eyes using 27G x ½” BD PrecisionGlide needles (depicted in Fig. 3A&B). The injections were placed 4 mm posterior to the limbus for phakic eyes and 3.5 mm posterior to the limbus for pseudophakic eyes. The force applied to the plunger of a syringe, hereafter referred to as the injection force, was measured. No significant difference in injection force was observed between syringe types when injected with the 15 cP mimic solution (Figure S2). However, when the 120 cP mimic solution was injected, the injection force observed while utilizing the BD and ClearJect syringes continually increased throughout the duration of the injection (Fig. 3C), while the injection force remained stable throughout the injection for StaClear and Zero Residual syringes. BD and ClearJect syringes demonstrated a 3.2-fold higher mean (Fig. 3D) and maximum (Fig. 3E) injection force compared to the Zero Residual and StaClear syringes. To assess whether injection force was dependent on the syringe type or a confounding variable, an ANOVA was performed on the full dataset (visualised in Fig. 3 & S2) using additional variables that were recorded during the experiment, including lens status, pre-injection IOP, IOP change, eye ID, injection duration, fluid density, and syringe type. As expected, fluid viscosity significantly impacted mean injection force (*p* = 0.0067), indicating that higher viscosity solutions require greater injection force. Notably, syringe type was the only other variable with a significant effect (*p* < 0.0001), supporting the hypothesis that syringe design influences injection force, with StaClear and Zero Residual outperforming BD and ClearJect syringes.

### Chemical stability (SE-HPLC)

**Fig. 4:**
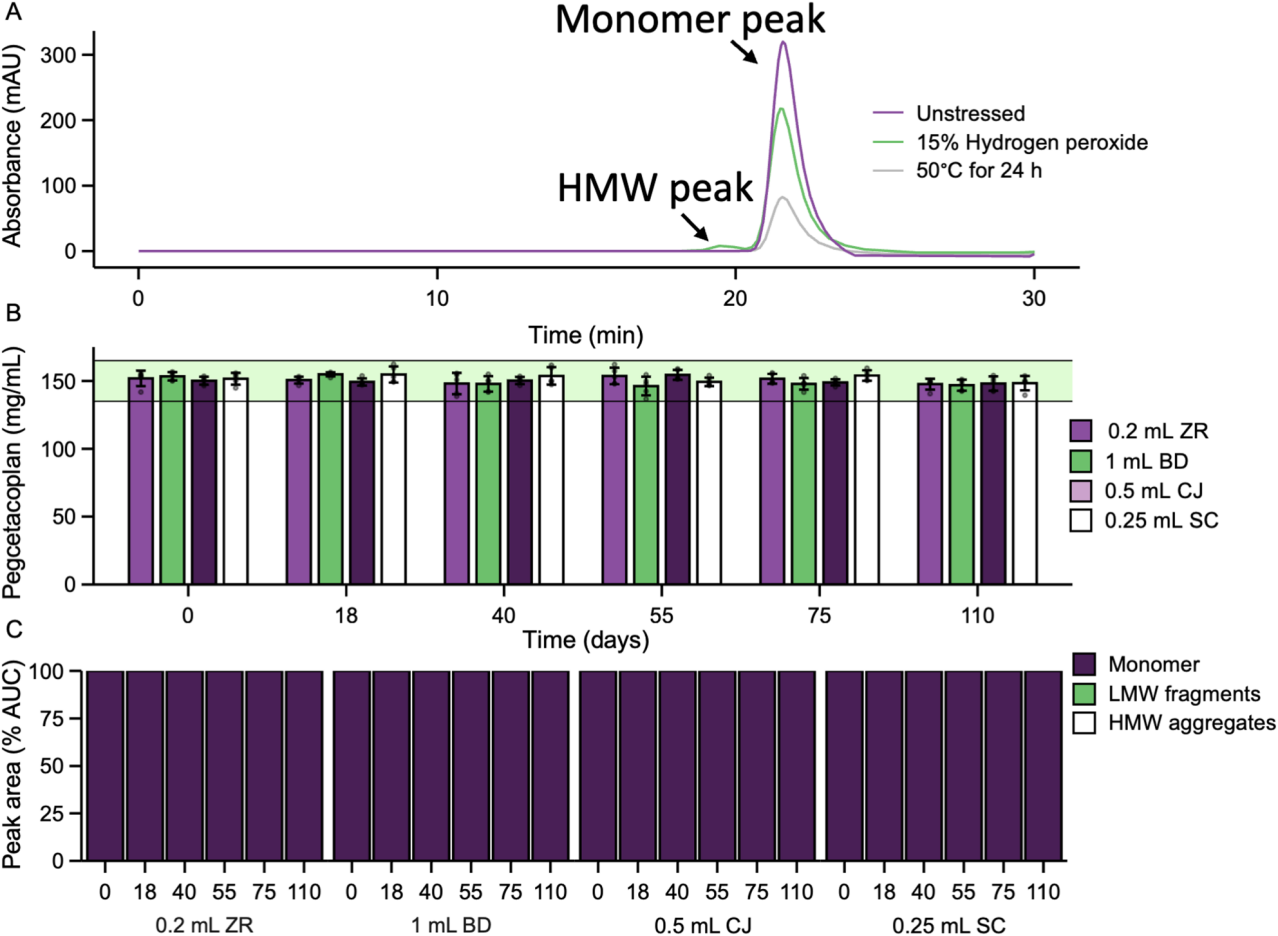
Potency of pegcetacoplan in various syringes. SYFOVRE (150 mg/mL pegcetacoplan) was compounded into 0.2 mL Zero Residual (ZR), 1 mL BD Luer-Lock (BD), 0.25 mL StaClear (SC), and 0.5 mL ClearJect (CJ) syringes and stored at 2–8 °C (protected from light) until the day of analysis. Within 5 days of the indicated timepoint, the potency of pegcetacoplan was assayed using size exclusion-high performance liquid chromatography (SE-HPLC). (**A**) Chromatogram of pegcetacoplan that was unstressed, subjected to 50 °C for 24 h, or treated with 15% hydrogen peroxide for 5 min. (**B**) Potency of pegcetacoplan compounded into various syringes over time. The shaded green area denotes the acceptable potency range in accordance with USP [32]. Bars represent the mean, error bars depict the standard deviation, and individual data points are shown as black dots. No significant difference was detected between syringes or between timepoints (ANOVA, Tukey’s HSD, p-value < 0.05). (**C**) Distribution of peaks corresponding to monomeric pegcetacoplan, higher molecular weight aggregates and lower molecular weight fragments based on percent of total area under the curve

A stability-indicating method of pegcetacoplan potency quantification was developed using size exclusion-high performance liquid chromatography (SE-HPLC). Detection of pegcetacoplan stability was assessed using forced degradation where pegcetacoplan samples were incubated at 50 °C for 24 h or exposed to 15% (v/v) hydrogen peroxide for 5 min and compared to an untreated control. Untreated pegcetacoplan had a single peak with a retention time of 21.5 min, representing the monomeric form of pegcetacoplan (Fig. 4A). Samples that underwent temperature- and oxidative-stress had the same monomeric peak at 21.5 min (Fig. 4A), but the area under the peak was smaller compared to unstressed samples (Figure S3) indicating degradation. Additionally, hydrogen peroxide-treated samples displayed a higher molecular weight peak at 19.5 min and a lower molecular weight peak at 23.5 min (Fig. 4A). To determine if the monomeric and higher molecular weight peaks had a similar chemical composition, stressed pegcetacoplan samples were analyzed via HPLC and the absorbance spectra for the main monomeric, high molecular weight (lower retention time), and lower molecular weight (higher retention time) peaks were compared. As expected, absorbance spectra of the lower molecular weight peak did not overlap with the monomeric peak (Figure S4), suggesting that this peak was made up of a different chemical composition, likely degradants of pegcetacoplan. On the other hand, the absorbance spectra of the high molecular weight and monomeric peaks significantly overlapped suggesting that the higher molecular weight peak contains intact molecules of pegcetacoplan. Since it is difficult to determine the predicted peak time for pegylated proteins on SEC-HPLC [31], this higher molecular weight species could represent a dimer, trimer, or higher-order oligomer of the monomer. The method was also validated for precision, accuracy, linearity, specificity, and robustness, in accordance with ICH Q2(R1) guidelines.

Pegcetacoplan consists of two biologically active, cyclic 15-amino acid peptides covalently linked to a PEG-899 molecule. Cleavage of either peptide (~ 1.3 kDa) would compromise pharmacostability and potentially cause a subtle shift in SE-HPLC retention time. However, no overlapping peaks, peak purity changes, or secondary peaks indicative of degradation were observed by HPLC during forced degradation. Therefore, SDS-PAGE was employed as an orthogonal method to confirm the structural integrity of the peptide–PEG linkage. Samples analyzed at day 56 showed no detectable lower molecular weight band (~ 1.3 kDa), confirming that no peptide cleavage had occurred and supporting the validation of the SE-HPLC method (Figure S5).

To assess protein stability, the potency of monomeric pegcetacoplan was monitored in the four syringe types over 110 days when stored at 2–8 °C, including a 24-hour temperature stress test at the end of the study period. In accordance with USP [32], pegcetacoplan potency remained within ± 10% of label potency over the course of the study period regardless of the syringe type used for storage (Fig. 4B), suggesting that pegcetacoplan remains stable under specified conditions for up to 110 days. Similarly, no protein aggregates or fragments were observed in any of the test samples at any time point in the experiment (Fig. 4C), suggesting that pegcetacoplan maintains strong physicochemical stability throughout the study period.

### Protein binding

**Fig. 5:**
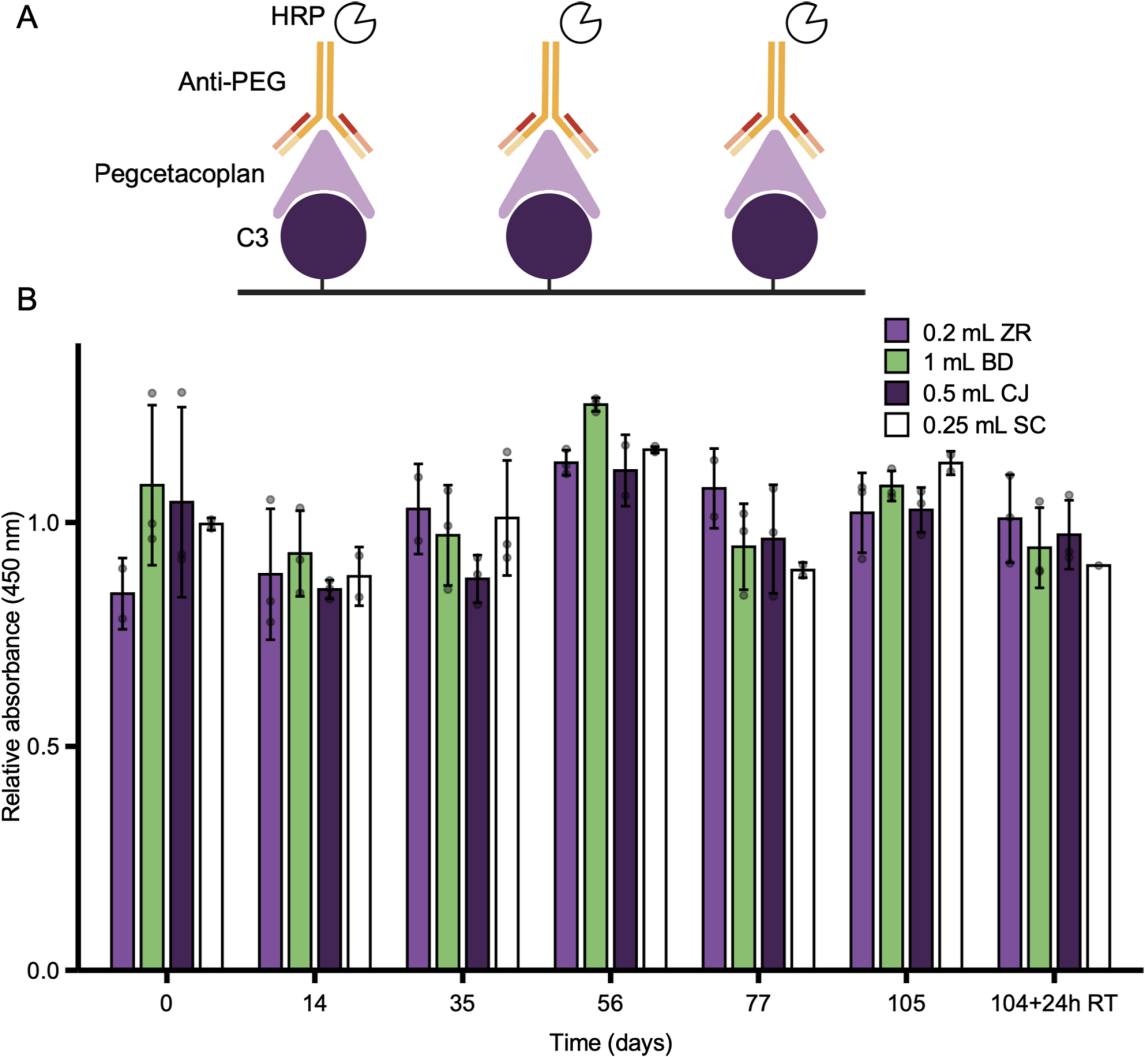
Pegcetacoplan binding stability to complement component C3. (**A**) Diagram of ELISA protein sandwich. (**B**) Protein binding stability of pegcetacoplan over time in various syringe types. Pegcetacoplan was retrieved from 0.2 mL Zero Residual (ZR), 1 mL BD Luer-Lock (BD), 0.25 mL StaClear (SC), or 0.5 mL ClearJect (CJ) at the indicated timepoint and diluted down to a concentration in the middle of the exponential curve (5 ng/mL). After 104 days a subset of samples was exposed to a 24-hour room temperature (19 to 24 °C) excursion. Relative absorbance was calculated by normalising values to the expected absorbance based on the standard curve (5-parameter logistic) generated during each assay. Bars represent the mean, error bars represent the standard deviation, and black dots represent the individual data points. No significant difference was observed between timepoints (ANOVA, Tukey’s HSD, p-value < 0.05)

The therapeutic efficacy of pegcetacoplan as a treatment for AMD is directly linked to its ability to bind complement component C3 and attenuate complement activation. To evaluate whether compounding or storage of pegcetacoplan in different syringe types impacted its binding capability, an ELISA was used to monitor C3 binding over time. Complement C3 was immobilized on a microplate, exposed to pegcetacoplan, and bound pegcetacoplan was detected using an anti-PEG antibody and HRP-conjugated secondary detection (depicted in Fig. 5A). Samples were collected from each syringe type at the specified timepoints and diluted to 5 ng/mL, which was in the linear portion of the standard curve. The binding activity of pegcetacoplan remained consistent throughout the 110-day study period, including after the 24-hour room temperature excursion (Fig. 5B), indicating that pegcetacoplan binding was not affected by syringe type.

### Other stability methods

In addition to molecular analyses, complementary methods were used to characterise the stability of SYFOVRE across different syringe types. Physical stability was evaluated through visual inspection for changes in color, phase separation, turbidity, and visible particulate contamination. No changes were observed throughout the 110-day study, indicating that SYFOVRE remained physically stable. The sterility of the preparation was tested as per USP <71> using membrane filtration in the middle and at the conclusion of the 110-day study period. SYFOVRE remained sterile throughout the study across all syringe types, confirming that container closures effectively prevented microbial ingress. Container closure integrity testing was also performed after 110 days using a 5 ± 2 μm laser-drilled defect as a positive control. All syringe types passed integrity testing (Figure S6), demonstrating maintenance of an effective barrier. The pH of SYFOVRE in the various syringes was also examined with no significant change over the course of the study (Figure S7).

## Discussion

The selected syringes were made from different manufacturing materials (borosilicate and polypropylene [PP]) with varying SiO content. The selection included a historically popular syringe (BD [PP] – SiO spray), two syringes with IVI indication (StaClear [PP] – Immobilized SiO & Zero Residual [PP] – SiO-free), and a unique low volume glass syringe (ClearJect [borosilicate] – Baked SiO).

Across all timepoints, including a 24-hour room temperature excursion at 110 days, pegcetacoplan maintained protein integrity, stability and epitope binding in all syringe types tested. This stability likely arises from PEG-induced thermostability [33] and stable structural features such as cysteine bonds that contribute to cyclization [34]. The biotherapeutic compatibility of the Zero Residual and BD syringes with SYFOVRE is consistent with previous reports demonstrating compatibility for the BD syringe with both Avastin [27] and Eylea [35], and compatibility for the Zero Residual syringe with Eylea, Avastin, Lucentis [29], and Vabysmo [30]. To our knowledge, this is the first study to comprehensively demonstrate biotherapeutic stability in ClearJect or StaClear syringes.

Given the confined, avascular, and non-regenerative nature of the vitreous, repeated IVIs require low particulate loads, especially in light of reported complications linked to SiO and other contaminants. In this study, the ClearJect borosilicate syringe with baked-on SiO exceeded the particulate limits as defined by USP <789>. Similarly, Krayukhina et al. (2014) observed that borosilicate syringes lubricated with SiO had significantly higher particulate counts than SiO-free polymer syringes (cyclic olefin polymer) [36]. This elevated particulate burden in borosilicate syringes may be linked to tungsten leaching, which has been connected to protein aggregation and immunogenicity [24] or SiO particulate contamination, that has been linked to protein aggregation [8, 37], immune activation [9–11], and vitreous SiO accumulation [4–7]. These findings highlight the critical importance of syringe material and design to minimize particulate contamination of ophthalmic preparations.

Injectability directly impacts IVI efficiency by affecting the force required for injection and physician workflow. Injectability decreases with formulation-driven increases in viscosity. Refrigeration predictably increases viscosity in Avastin, Eylea, Vabysmo, and SYFOVRE, where SYFOVRE showed a 38% increase (Table S1), reinforcing the need for room temperature equilibration prior to injection. Our injectability studies demonstrated that conventional BD and ClearJect syringes required significantly higher injection force for higher viscosity formulation mimics as compared to purpose-built low-volume syringes (StaClear and Zero Residual), which maintained smoother and more consistent injection profiles. The longer stroke lengths in StaClear and Zero Residual syringes also removed the injection force spike observed in BD and ClearJect syringes.

While this study enhances our understanding of syringe performance in the context of IVIs, a few limitations in the experimental design should be acknowledged. Most studies measuring properties of the vitreous do so at 37 °C to mimic physiological conditions [38], while this study used cadaver eyes equilibrated to room temperature. This lower temperature could decrease the intravitreal fluid viscosity and potentially lead to higher observed injection force compared to similar studies conducted at 37 °C. However, the viscosity of intravitreal fluid is more complex than Newtonian fluids like water because it exhibits the non-Newtonian behavior of shear-thinning. Shear-thinning means that above a limit of shear stress the viscosity of the fluid decreases. In the case of intravitreal fluid, at shear stresses greater than 0.2 Pa its viscosity drops 1,000,000-fold to approximately the viscosity of water [39]. Notably the 0.2 Pa threshold is less than the 6.7–80.7 Pa retinal pigment epithelium stress measured during a simulated ophthalmic injection [40]. Additionally, Silva et al. (2017), observed that there were no significant differences in the viscosity of intravitreal fluid at 37 °C compared to 20 °C across a range of shear stresses. Taken together, this data suggests that the injection dynamics observed would not be significantly impacted by this temperature difference. More importantly for this study, all experiments were conducted under identical temperature conditions; therefore, comparisons of injectability between injection devices are valid despite a potential temperature-related shift in vitreous viscosity. This study used a randomized selection of cadaveric eyes provided anonymously from the Eye Bank of Canada. The eyes likely covered a wide range of ocular statuses present in real-world clinical settings (lens status and vitreous intactness) but were likely skewed older due to being sourced from deceased individuals, which could influence injection dynamics. To avoid lens status becoming a confounding variable in injection force, eyes with different lens statuses were distributed evenly amongst different syringe types (summarised in Table S2). As this study is not a clinical trial, the conclusions drawn from the injection force experiment may not translate directly into in vivo performance. Finally, the SYFOVRE stability study assumed ideal compounding conditions (i.e. careful handling and no mechanical stress from shipment) which may not represent real world conditions.

This study is unique in that it independently assessed both syringe-associated protein therapeutic compatibility and in-use practicality, providing a comprehensive understanding of syringe suitability from both analytical and operational perspectives. We provide foundational data to guide syringe selection for SYFOVRE and other high viscosity formulations, offering insights to support the administration of the first approved treatment for dry AMD. This study provides evidence that the syringes designed for ophthalmic injections, StaClear and Zero Residual, outperformed standard syringes in injectability and matched or exceeded standard syringe performance in particulate burden, drug stability, and sterility. These findings are broadly applicable to biologic therapeutics spanning a wide range of viscosities and provide guidance for syringe selection to aid effective IVI therapeutic administration.

Access to syringes specifically tailored for intravitreal injection is now expanding, providing ophthalmologists with safer and more consistent delivery options. For example the Zero Residual and StaClear syringes are currently available in the United States, Canada, and across Europe. For high-cost pharmacotherapies such as pegcetacoplan (SYFOVRE), standard Luer-lock syringes can retain a clinically relevant dead-space volume [41] so minimizing drug waste, through use of medical devices with minimal dead space like the Zero Residual syringe, is operationally important and resource efficient. This is particularly beneficial for compounding pharmacies aiming to maximize dosing efficiency from single-use vials.

While regulatory agencies evaluate performance data to support syringes being labelled for ophthalmic use, there are no globally harmonized standards establishing uniform acceptance criteria. As a result, the burden of selecting ophthalmic-specific syringes for patients largely falls on the ophthalmology community, and standards vary from clinic to clinic. Instead, global regulatory standards should be established in consultation with the ophthalmology community that place the evidentiary burden on manufacturers to demonstrate ophthalmic safety and performance. While existing ISO standards such as ISO 7886 and ISO 11040 define baseline mechanical and dimensional requirements for syringes, they do not address the unique risks associated with intraocular delivery. Given the sensitive nature of the vitreous, ophthalmic-specific syringes should be required to meet pre-defined acceptance criteria directly linked to patient safety. We propose that a future standard should include the following parameters: dose accuracy within the clinically relevant 50–100 µL range, injection force profile, intraocular pressure spikes in animal cadaver eyes, particulate shedding into solution, and silicone oil entrainment in solutions spanning a range of viscosities. Establishing standardized, regulator-endorsed test methods and predefined acceptance criteria in collaboration with the ophthalmology community would shift syringe qualification from observation-based ophthalmologist-specific preference to evidence-based certification, thereby reducing variability in clinical practice and ensuring that devices intended for intravitreal use are demonstrably fit for purpose before reaching patients. This would allow the standards to evolve over time, leading to continual improvement in ophthalmic syringe technology.

## Electronic Supplementary Material

Below is the link to the electronic supplementary material.

Supplementary Material 1: Figure S1. Photos of the syringes used in this study post fluid expression. 0.2 mL Zero Residual (ZR), 1 mL BD Luer-Lock (BD), 0.25 mL StaClear (SC), and 0.5 mL ClearJect (CJ) were held under an LED light and photographed. The white arrow depicts residue build up.

Supplementary Material 2: Figure S2. Injection force of various syringes for 15 cP solution delivered into human cadaver eye vitreous. 0.2 mL Zero Residual (ZR), 1 mL BD Luer-Lock (BD), 0.25 mL StaClear (SC), and 0.5 mL ClearJect (CJ) syringes were compounded with either 0.12 mL of a 15 cP (above) or 120 cP (Fig. 3) viscosity mimic solution. A BD 27G x ½” precision glide needle was affixed, and the device was primed to 0.1 mL prior to injection into the vitreous of human cadaver eyes. All injections were performed by the same ophthalmologist. (A) Injection force over time of various syringes filled with 15 cP mid range viscosity mimic solution. Dots represent the mean injection force at 0.5 s intervals. The lines represent a non-linear regression fit for injection force over time for each syringe type. The mean (D) and max (E) injection force for intravitreal injections. Bars represent the aggregate average of the mean and max injection forces for multiple injections and error bars indicate the standard deviation. Dots represent the mean injection force for individual injections. Different letters indicate statistically significant differences (ANOVA, Tukey’s HSD, p-value < 0.05).

Supplementary Material 3: Figure S3. Potency of pegcetacoplan after exposure to various stress conditions. SYFOVRE (150 mg/mL pegcetacoplan) was either unstressed, treated with 15% hydrogen peroxide for 5 min, or subjected to 50 °C for 24 h. (A) Potency of pegcetacoplan when SYFOVRE was exposed to various stressors. Bars represent the mean, error bars depict the standard deviation, and individual data points are shown as black dots. Different letters indicate significant difference (ANOVA, Tukey’s HSD, p-value < 0.05). (B) Percentage of peak area corresponding to monomeric pegcetacoplan, higher molecular weight (HMW) aggregates and lower molecular weight (LMW) fragments based on percent of total area under the curve.

Supplementary Material 4: Figure S4. Absorbance spectra of peaks observed in pegcetacoplan stressed with 15% hydrogen peroxide for 5 min.

Supplementary Material 5: Figure S5. SDS-PAGE of SYFOVRE from compounded syringes. SYFOVRE (150 mg/mL pegcetacoplan) was compounded into 0.2 mL Zero Residual (ZR), 1 mL BD Luer-Lock (BD), 0.25 mL StaClear (SC), and 0.5 mL ClearJect (CJ) syringes (experimental duplicate) and stored at 2–8 °C. After 56 days, the compounded solution from each syringe was collected and two samples were taken directly from a vial as a control. Samples were incubated under reducing condition then subjected to SDS-PAGE. Protein was visualised by treating samples with Coomassie dye G-250. A molecular weight marker (M) was run alongside samples and the bands are annotated with their molecular weight. The black arrow indicates the gel front.

Supplementary Material 6: Figure S6. Container closure integrity testing. SYFOVRE (150 mg/mL pegcetacoplan) was compounded into 0.2 mL Zero Residual (ZR), 1 mL BD Luer-Lock (BD), 0.25 mL StaClear (SC), and 0.5 mL ClearJect (CJ) syringes (experimental duplicate) and stored at 2–8 °C. After 110 days, syringes were subjected to container closure integrity testing via a methylene blue dye ingress method in accordance with USP <1207>. The bars represent the mean, and error bars depict the standard deviation. Different letters indicate significant differences (ANOVA, Tukey’s HSD, p-value < 0.05).

Supplementary Material 7: Figure S7. pH of SYFOVRE in various syringes over time. SYFOVRE was compounded into 0.2 mL Zero Residual™ (ZR), 1 mL BD Luer-Lock™ (BD), 0.25 mL StaClear (SC), and 0.5 mL ClearJect (CJ) and stored at 2–8 °C until the day of analysis. The pH of SYFOVRE was assayed within 5 days of the indicated timepoint. The bars represent the mean, error bars depict the standard deviation, and individual data points are shown as black dots. No significant difference was detected between syringes or between timepoints (ANOVA, Tukey’s HSD, p-value < 0.05).

Supplementary Material 8: Figure S8. Mid-range injection force for different needles used in intravitreal injections. Injection force of 0.2 mL Zero Residual syringes with various Luer-Lock needle attachments. Syringes were compounded with 0.12 mL of a 15 cP viscosity mimic. The indicated needle was affixed, and the device was primed to 0.1 mL prior to mock injection. All mock injections were performed by the same individual. Bars represent the aggregate average of the mean injection force for multiple injections and error bars indicate the standard deviation. Different letters indicate statistically significant differences (ANOVA, Tukey’s HSD, p-value < 0.05).

Supplementary Material 9: Table S1. Viscosity of Avastin, Eylea, Vabysmo, and SYFOVRE at room temperature and refrigerated.

Supplementary Material 10: Table S2. Unaggregated eye characteristics and raw injection force data.

Supplementary Material 11. Supplementary methods.

## Data Availability

Study data is not publicly available. Contact the authors if required.

## Declarations

### Brief summary statement

Pegcetacoplan (SYFOVRE), the first FDA-approved therapy for dry AMD supplied only in vial format, requires careful syringe selection due to its high viscosity. In this study, stability was preserved across syringe types, but injectability and particulate burden varied. Ophthalmic-specific syringes, especially Zero Residual and StaClear, outperformed conventional syringes, supporting safer, more efficient intravitreal delivery.
