# Supplementary figures and images for: Guiding syringe selection for intravitreal injections: injectability and stability analysis of compounded pegcetacoplan (SYFOVRE) and the broader implications for high-viscosity ophthalmic therapies
Source: Int J Retina Vitreous. 2026 Mar 12;12:63. doi: 10.1186/s40942-026-00832-3 (PMC13147785; doi:10.1186/s40942-026-00832-3)

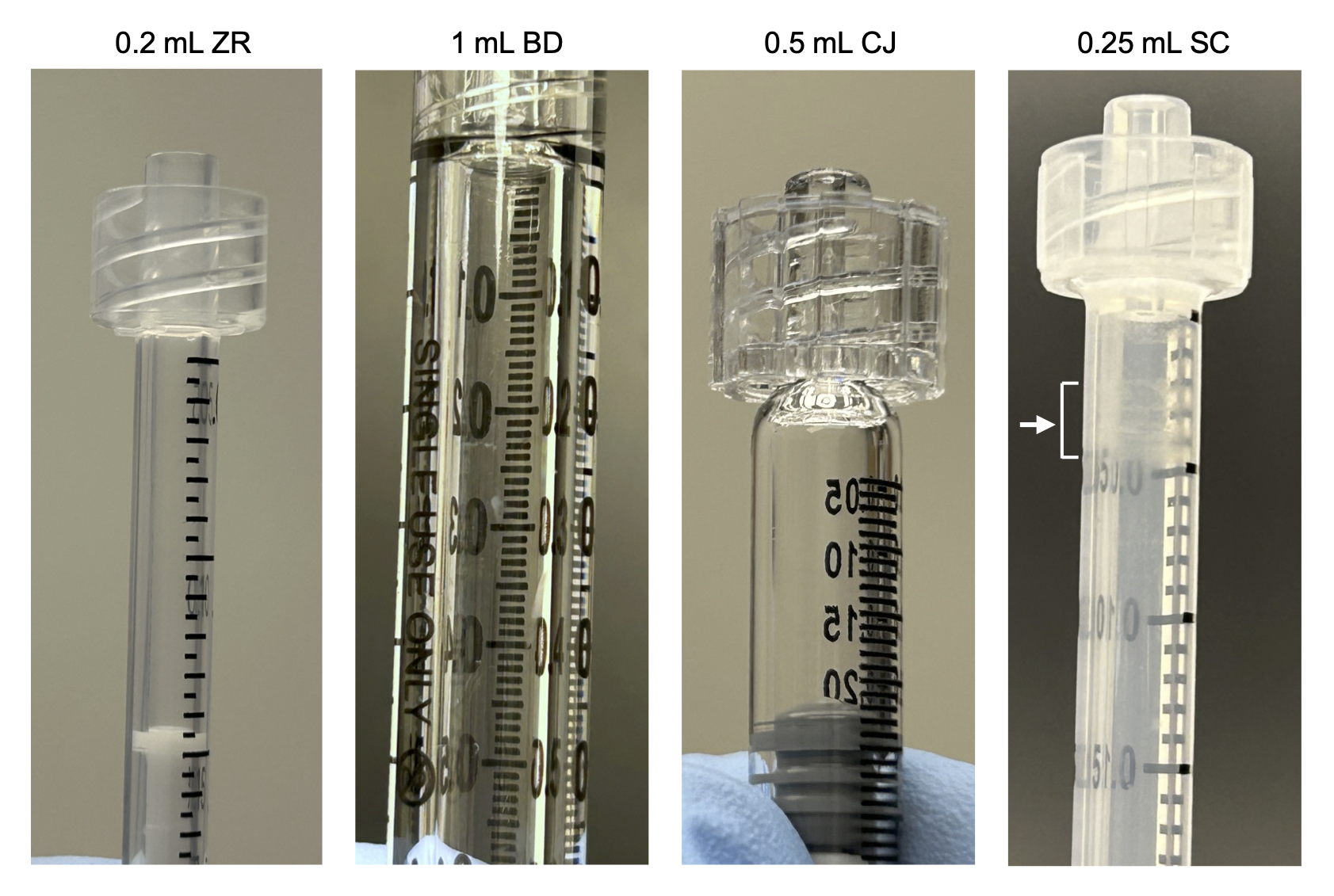

Supplement: Supplementary file 1 — Supplementary Material 1: Figure S1. Photos of the syringes used in this study post fluid expression. 0.2 mL Zero Residual (ZR), 1 mL BD Luer-Lock (BD), 0.25 mL StaClear (SC), and 0.5 mL ClearJect (CJ) were held under an LED light and photographed. The white arrow depicts residue build up. [file 40942_2026_832_MOESM1_ESM.png]

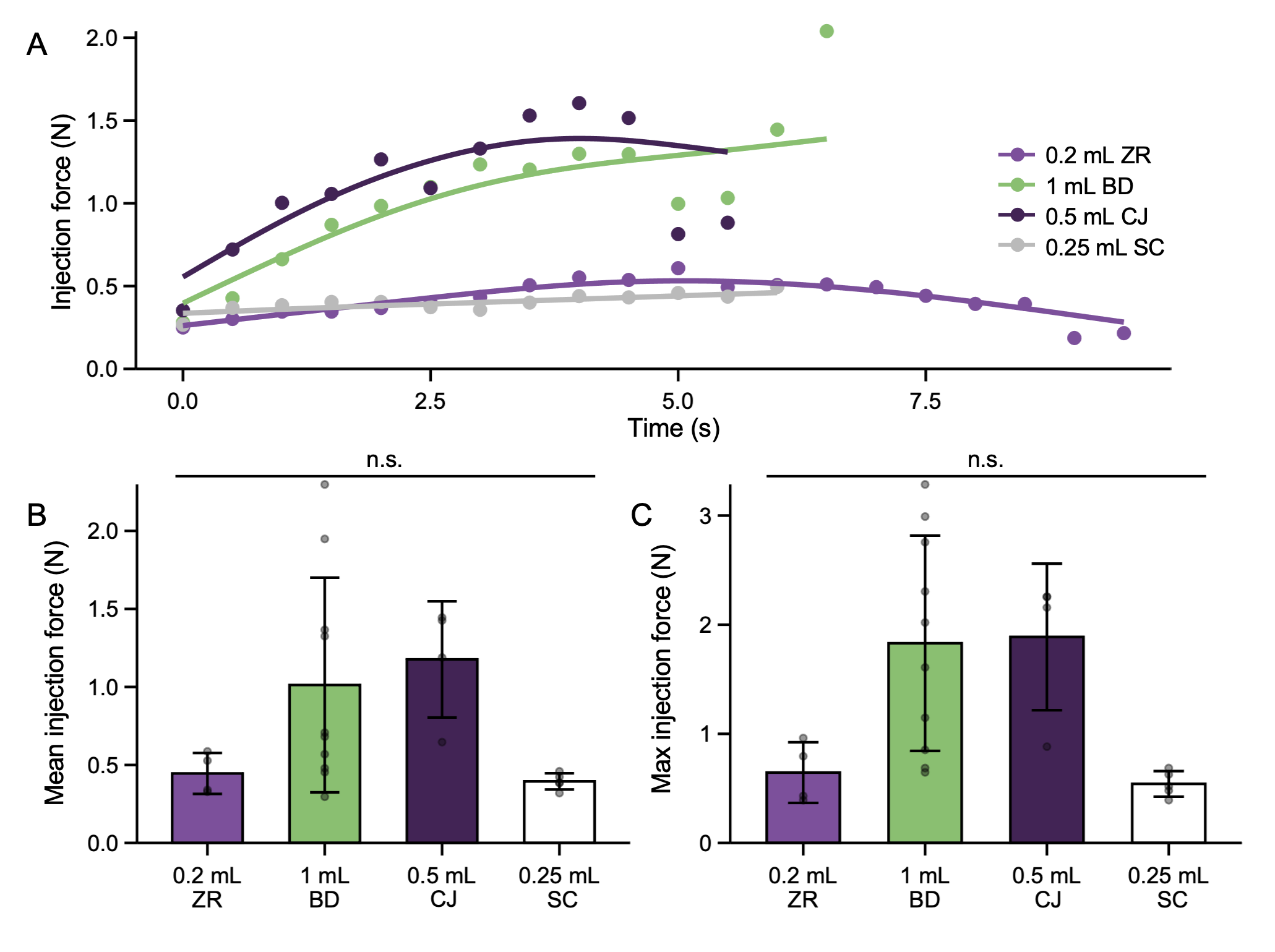

Supplement: Supplementary file 2 — Supplementary Material 2: Figure S2. Injection force of various syringes for 15 cP solution delivered into human cadaver eye vitreous. 0.2 mL Zero Residual (ZR), 1 mL BD Luer-Lock (BD), 0.25 mL StaClear (SC), and 0.5 mL ClearJect (CJ) syringes were compounded with either 0.12 mL of a 15 cP (above) or 120 cP (Fig. 3) viscosity mimic solution. A BD 27G x ½” precision glide needle was affixed, and the device was primed to 0.1 mL prior to injection into the vitreous of human cadaver eyes. All injections were performed by the same ophthalmologist. (A) Injection force over time of various syringes filled with 15 cP mid range viscosity mimic solution. Dots represent the mean injection force at 0.5 s intervals. The lines represent a non-linear regression fit for injection force over time for each syringe type. The mean (D) and max (E) injection force for intravitreal injections. Bars represent the aggregate average of the mean and max injection forces for multiple injections and error bars indicate the standard deviation. Dots represent the mean injection force for individual injections. Different letters indicate statistically significant differences (ANOVA, Tukey’s HSD, p-value < 0.05). [file 40942_2026_832_MOESM2_ESM.png]

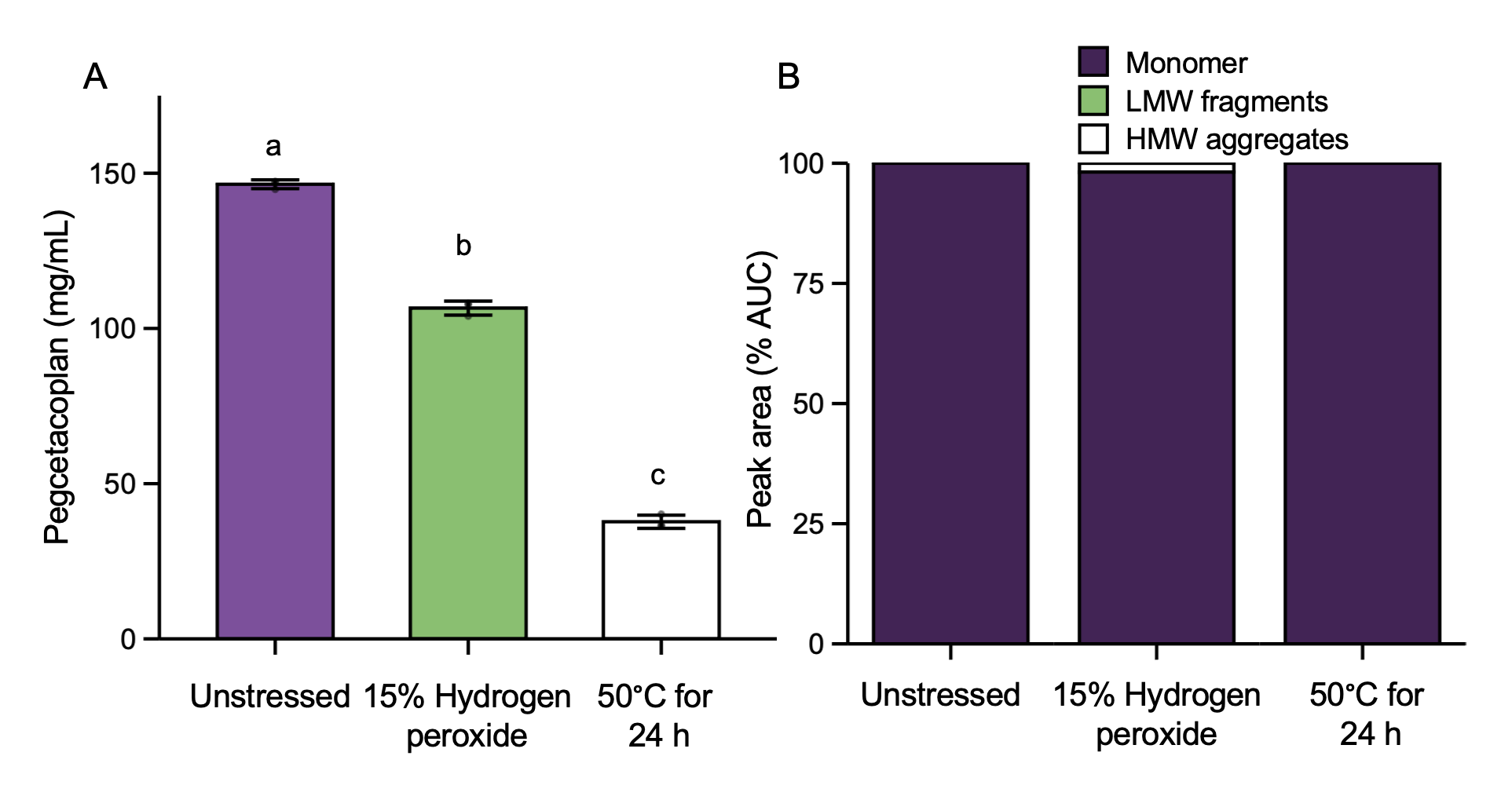

Supplement: Supplementary file 3 — Supplementary Material 3: Figure S3. Potency of pegcetacoplan after exposure to various stress conditions. SYFOVRE (150 mg/mL pegcetacoplan) was either unstressed, treated with 15% hydrogen peroxide for 5 min, or subjected to 50 °C for 24 h. (A) Potency of pegcetacoplan when SYFOVRE was exposed to various stressors. Bars represent the mean, error bars depict the standard deviation, and individual data points are shown as black dots. Different letters indicate significant difference (ANOVA, Tukey’s HSD, p-value < 0.05). (B) Percentage of peak area corresponding to monomeric pegcetacoplan, higher molecular weight (HMW) aggregates and lower molecular weight (LMW) fragments based on percent of total area under the curve. [file 40942_2026_832_MOESM3_ESM.png]

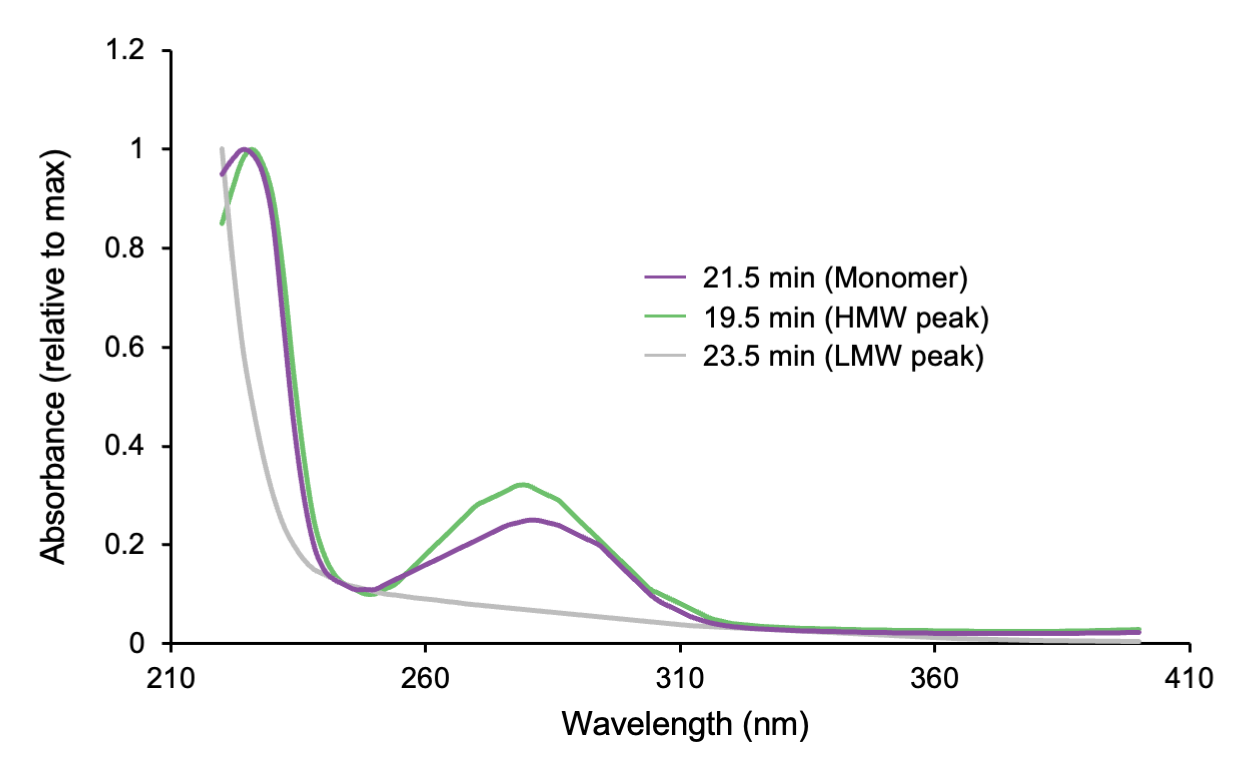

Supplement: Supplementary file 4 — Supplementary Material 4: Figure S4. Absorbance spectra of peaks observed in pegcetacoplan stressed with 15% hydrogen peroxide for 5 min. [file 40942_2026_832_MOESM4_ESM.png]

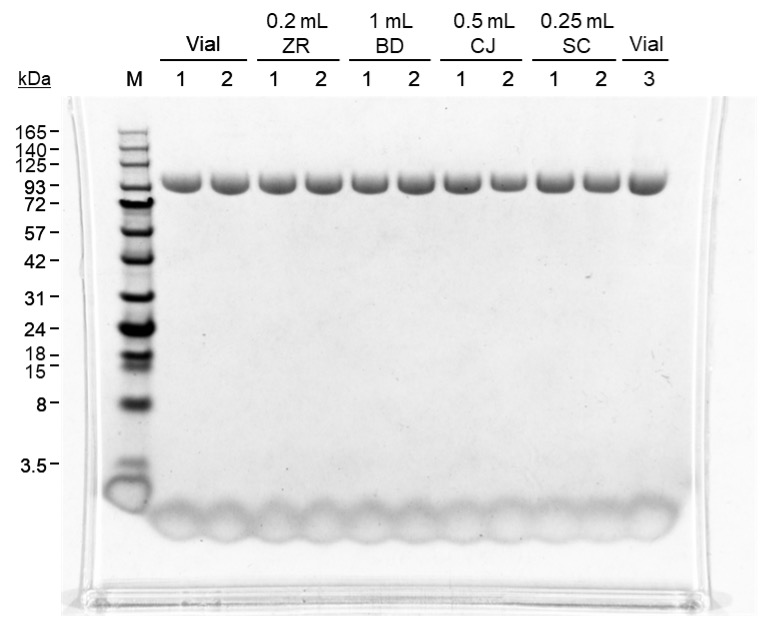

Supplement: Supplementary file 5 — Supplementary Material 5: Figure S5. SDS-PAGE of SYFOVRE from compounded syringes. SYFOVRE (150 mg/mL pegcetacoplan) was compounded into 0.2 mL Zero Residual (ZR), 1 mL BD Luer-Lock (BD), 0.25 mL StaClear (SC), and 0.5 mL ClearJect (CJ) syringes (experimental duplicate) and stored at 2–8 °C. After 56 days, the compounded solution from each syringe was collected and two samples were taken directly from a vial as a control. Samples were incubated under reducing condition then subjected to SDS-PAGE. Protein was visualised by treating samples with Coomassie dye G-250. A molecular weight marker (M) was run alongside samples and the bands are annotated with their molecular weight. The black arrow indicates the gel front. [file 40942_2026_832_MOESM5_ESM.png]

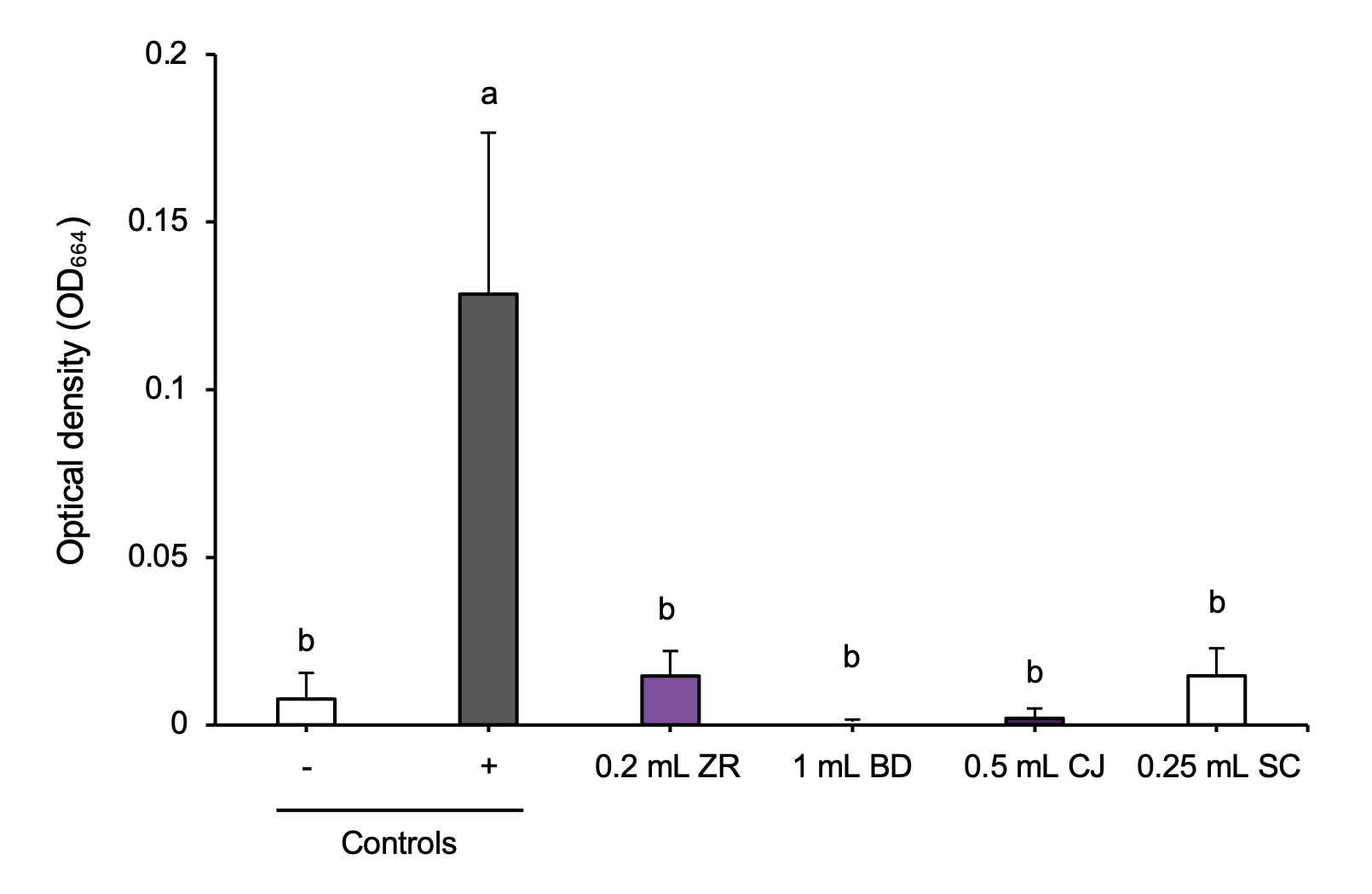

Supplement: Supplementary file 6 — Supplementary Material 6: Figure S6. Container closure integrity testing. SYFOVRE (150 mg/mL pegcetacoplan) was compounded into 0.2 mL Zero Residual (ZR), 1 mL BD Luer-Lock (BD), 0.25 mL StaClear (SC), and 0.5 mL ClearJect (CJ) syringes (experimental duplicate) and stored at 2–8 °C. After 110 days, syringes were subjected to container closure integrity testing via a methylene blue dye ingress method in accordance with USP <1207>. The bars represent the mean, and error bars depict the standard deviation. Different letters indicate significant differences (ANOVA, Tukey’s HSD, p-value < 0.05). [file 40942_2026_832_MOESM6_ESM.png]

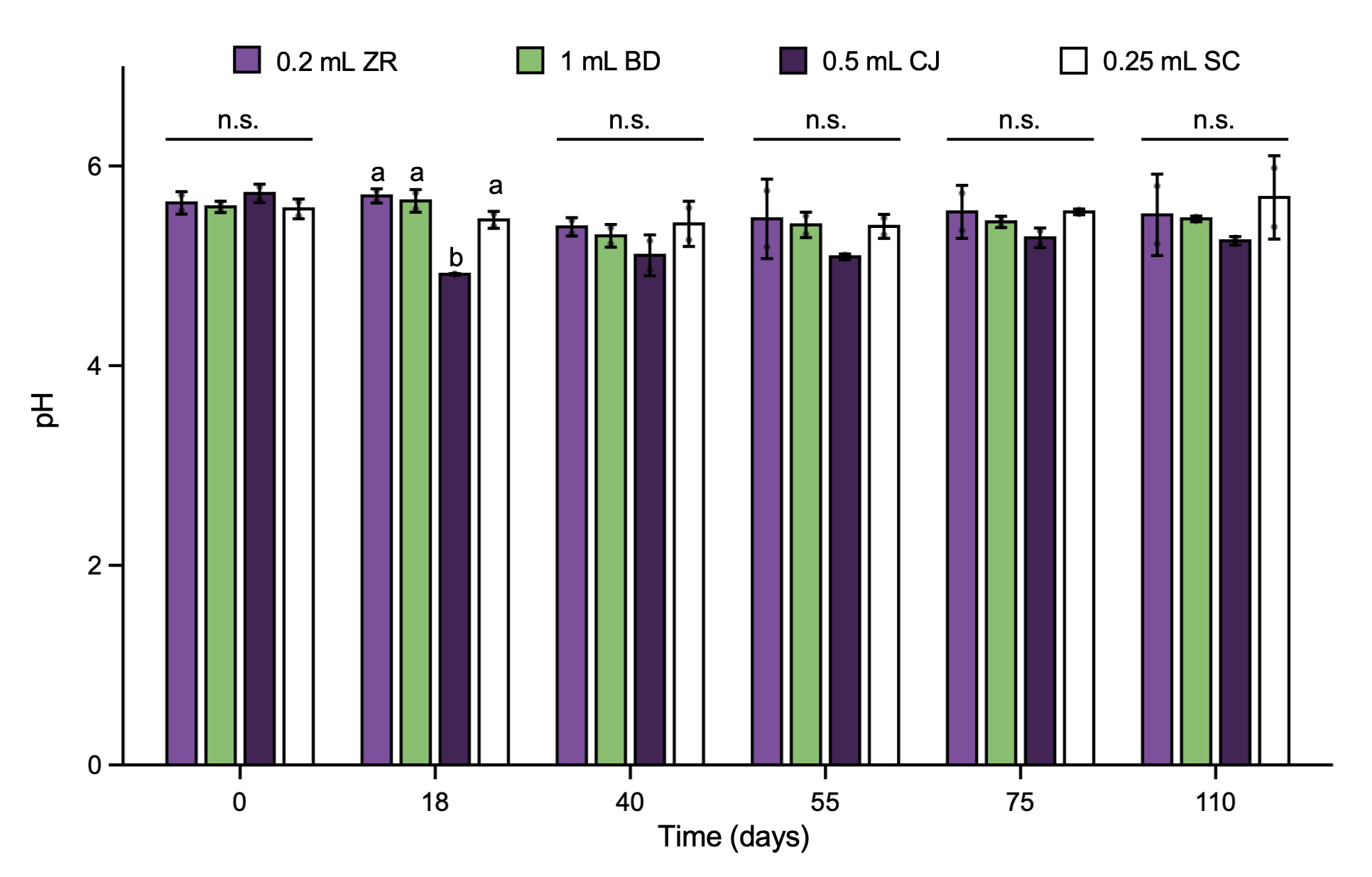

Supplement: Supplementary file 7 — Supplementary Material 7: Figure S7. pH of SYFOVRE in various syringes over time. SYFOVRE was compounded into 0.2 mL Zero Residual™ (ZR), 1 mL BD Luer-Lock™ (BD), 0.25 mL StaClear (SC), and 0.5 mL ClearJect (CJ) and stored at 2–8 °C until the day of analysis. The pH of SYFOVRE was assayed within 5 days of the indicated timepoint. The bars represent the mean, error bars depict the standard deviation, and individual data points are shown as black dots. No significant difference was detected between syringes or between timepoints (ANOVA, Tukey’s HSD, p-value < 0.05). [file 40942_2026_832_MOESM7_ESM.png]

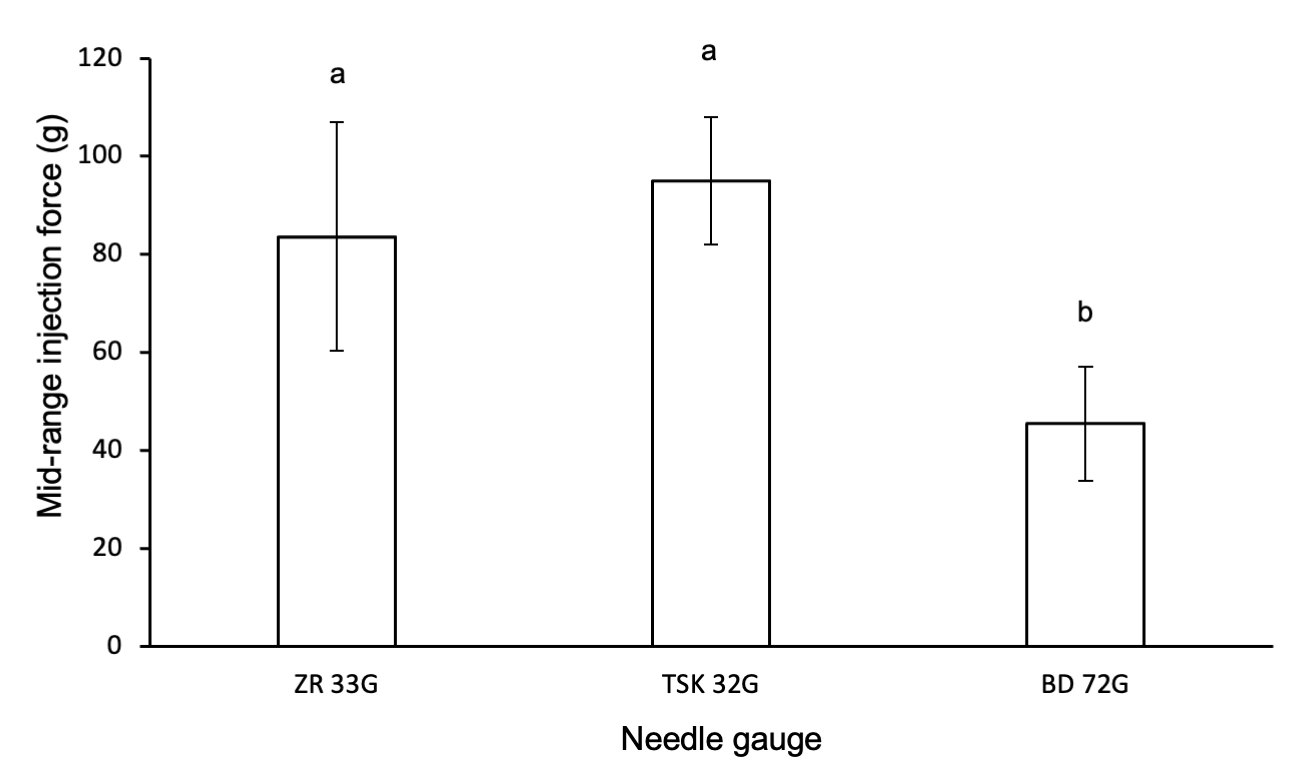

Supplement: Supplementary file 8 — Supplementary Material 8: Figure S8. Mid-range injection force for different needles used in intravitreal injections. Injection force of 0.2 mL Zero Residual syringes with various Luer-Lock needle attachments. Syringes were compounded with 0.12 mL of a 15 cP viscosity mimic. The indicated needle was affixed, and the device was primed to 0.1 mL prior to mock injection. All mock injections were performed by the same individual. Bars represent the aggregate average of the mean injection force for multiple injections and error bars indicate the standard deviation. Different letters indicate statistically significant differences (ANOVA, Tukey’s HSD, p-value < 0.05). [file 40942_2026_832_MOESM8_ESM.png]
