# Supplementary material for: Guiding syringe selection for intravitreal injections: injectability and stability analysis of compounded pegcetacoplan (SYFOVRE) and the broader implications for high-viscosity ophthalmic therapies
Source: Int J Retina Vitreous. 2026 Mar 12;12:63. doi: 10.1186/s40942-026-00832-3 (PMC13147785; doi:10.1186/s40942-026-00832-3)
Supplement: Supplementary file 9 — Supplementary Material 9: Table S1. Viscosity of Avastin, Eylea, Vabysmo, and SYFOVRE at room temperature and refrigerated. [file 40942_2026_832_MOESM9_ESM.docx]

**Table S1.** Viscosity of Avastin, Eylea, Vabysmo, and SYFOVRE at room temperature and refrigerated.

| Product | Temperature (°C) | Viscosity (cP) |
| --- | --- | --- |
| Avastin | Room temperature (19-25°C) | 1.1 |
|  | Refrigerated (2-8°C) | 1.5 |
| Eylea | Room temperature (19-25°C) | 1.8 |
|  | Refrigerated (2-8°C) | 2.0 |
| Vabysmo | Room temperature (19-25°C) | 9.1 |
|  | Refrigerated (2-8°C) | 14.7 |
| SYFOVRE | Room temperature (19-25°C) | 118.0 |
|  | Refrigerated (2-8°C) | 163.7 |
