# Supplementary material for: Guiding syringe selection for intravitreal injections: injectability and stability analysis of compounded pegcetacoplan (SYFOVRE) and the broader implications for high-viscosity ophthalmic therapies
Source: Int J Retina Vitreous. 2026 Mar 12;12:63. doi: 10.1186/s40942-026-00832-3 (PMC13147785; doi:10.1186/s40942-026-00832-3)
Supplement: Supplementary file 11 — Supplementary Material 11. Supplementary methods. [file 40942_2026_832_MOESM11_ESM.docx]

Supplementary Methods

**Particulates Testing**

A total of 234 syringes per replicate of BD, Zero Residual, StaClear, and ClearJect syringes were compounded with 0.15 mL Sterile Water for Injection (Baxter Canada) in an ISO 5 laminar flow biosafety cabinet. Syringe contents were pooled in a particle-free flask and sonicated for 30 seconds. Particulate burden was then evaluated in technical triplicate using the HIAC 9703 liquid particle counter (Beckman Coulter) following USP <789> Particulate Matter in Ophthalmic Solutions: Light Obscuration Particle Count Test. Testing was conducted in experimental triplicate, with at least two independent syringe lots analyzed for each syringe type. The sterile water for injection was also tested for particulate matter as a control.

**Injectability Experiment:**

As specified in SYFOVRE monograph (Apellis, 2023), sterile 27-gauge PrecisionGlide ½ inch needles (BD, NJ, USA) were attached to four syringe types, each prefilled with 0.12 mL of viscosity mimic (15 cP or 120 cP). Syringes were primed to 0.10 mL and inspected for bubbles. Experiments were performed at room temperature (19–25°C). Anonymous human donor eyes were obtained from the Eye Bank of Canada. A bespoke apparatus was used to stabilize globes. To induce physiological IOP, a gentle negative pressure was applied to the back of the eye using a 60 mL syringe connected to silicone tubing affixed to the posterior globe. The negative pressure was adjusted until IOP was in normal physiological range. IOP was measured with a Reichert Tono-Pen**^®^** Avia (SKU 230650). The injections were placed 4 mm posterior to the limbus for phakic eyes and 3.5 mm posterior to the limbus for pseudophakic eyes.To minimize injector fatigue bias, procedures were split across two sessions with randomized syringe order. Each donor eye received a maximum of four injections. Injection pressure was recorded using a Garosa (5000g Max. Pressure Display Module) with sensing film on the injector’s thumb.

**Stability Study**

**SDS-PAGE**

SYFOVRE vial (Apellis) and T56 day syringe samples were run in experimental duplicate on sodium dodecyl-sulfate polyacrylamide gel electrophoresis (SDS-PAGE) under reducing conditions. Samples were diluted with nuclease free water (Sigma-Aldrich) and incubated with dithiothreitol (Fisher Scientific) SDS-loading Buffer for 5 minutes at 95°C. 5 µg of protein was loaded per well into a NuPAGE 4-16% Bis Tris Gel (Thermo Fisher Scientific) and then run for 75 minutes at 90 V. Bromophenol blue (BB) (Sigma-Aldrich) was added as a dye to the samples. BB has a size of ~0.7 kDa and is visualized as the dye front. BLUelf Prestained Protein Ladder (GeneDireX) was used for a size standard. Protein visualization was achieved through coomassie staining using Gel Code Blue Stain Reagent (Thermo Fisher Scientific). Gel was imaged using BioRad Gel Doc XR+ Imaging System.

Needle gauge affects needle lumen size and injection force, with larger-gauge (smaller diameter) needles requiring more force. For example, assuming the same wall thickness a 27 G needle will have a larger lumen diameter and therefore lower injection force than a comparable 33 G needle. Due to limited human cadaver eye availability, only one needle gauge could be selected to perform all the comparisons required for this study. Although higher gauge needles (33 to 30 G) have historically been used for lower viscosity intravitreal injections to facilitate ease of scleral penetration, a 27 G × ½” needle was selected because it was recommended for use in the SYFOVRE monograph and showed the lowest injection pressures in preliminary testing (Figure S8). As a result of the 27G exhibiting the lowest injection pressures, any statistically significant findings using the 27G needle would be expected to be amplified in smaller-lumen needles, making the comparisons performed in this study also applicable to higher gauge needles.

HPLC Size-Exclusion Chromatography

A stability-indicating SE-HPLC method for pegcetacoplan potency and aggregation measurements was developed in compliance with USP <1225> and ICH method validation parameters. Forced degradation analysis was performed to assess specificity and the stability indicating nature of the test methods. System suitability was conducted prior to each analysis. Contents from each syringe were transferred into a glass HPLC vial and 150 mg/mL SYFOVRE was then serially diluted to 1.5 mg/mL with mobile phase. Using the Agilent 1100 HPLC System (Agilent Technologies), 5 µL was injected into Superdex 200 Increase 3.2/300 column (Cytiva Life Sciences) with a flow rate of 0.050 mL/min and using 0.1 M potassium phosphate buffer (pH 6) (Sigma-Aldrich) mobile phase. Column temperature was set to 23 °C. UV Absorption was detected by diode array detector (DAD) at 222 nm. Each sample was measured in technical quadruplicate and at minimum experimental triplicate (N = 3 – 5).

**ELISA – C3 Binding**

The ELISA coating solution was prepared by diluting concentrated 1.07 mg/mL Complement C3, Human (Millipore Sigma) to a final concentration of 2 μg/mL C3 with ELISA phosphate coating buffer (Invitrogen). 96-well clear polystyrene plates (Thermo Scientific) were coated with 100 µL of the ELISA coating solution. Plates were incubated for 1 hour (h) at room temperature (RT), washed once with 300 µL 1X phosphate buffered saline (PBS) (Gibco), and blocked with 300 µL of 5% (w/v) skim milk in PBS (Medallion). Plates were stored overnight at 4°C, and the blocking buffer was aspirated the next day. Pegcetacoplan standards (Apellis) were serially diluted to a range of concentrations from 450,000–5.76 pg/mL in 1% (w/v) skim milk in PBS. Syringe samples were collected and diluted to 5 ng/mL in 1% (w/v) skim milk in PBS. Plates were incubated for 1 h at RT on an orbital shaker (200 rpm), then washed three times with 300 μL PBS. Biotinylated anti-PEG antibody (GenScript, 1 µg/mL) was added and incubated for 2 h at RT, followed by three washes and 1 h incubation with streptavidin-HRP (Thermo Fisher, 1:5,000). After three washes, 100 µL TMB substrate (Thermo Scientific) was added and incubated up to 30 min. Reactions were stopped with 25% acetic acid, and absorbance read at 450 nm.

**pH Measurements:**

SYFOVRE pH was measured using a Horiba LaquaTwin Model 22 pH probe with a resolution of 0.01 pH units. pH measurements were taken from each syringe in experimental duplicate. Tests were performed in compliance of USP <791>, the probe was calibrated on each testing day using a two-point calibration with pH 4.00 and 7.00 solutions prepared by PCI Scientific.

**Physical Stability (appearance):**

Physical stability of pegcetacoplan was determined on each study day by visual inspection for particulate matter as per USP <789> and changes in the colour, phase, separation, and turbidity as per USP <1149> and <1790> recommendations. Physical stability of the preparation was defined as no change in the visually inspected parameters.

**Microbiological Stability:**

USP <71> sterility testing was conducted via membrane filtration using Sterisart cannisters (Sartorius) and incubated with fluid thioglycolate medium (FTM) (bioMérieux) and tryptic soy broth (TSB) (bioMérieux). Method suitability was conducted in accordance with USP <71> to ensure that the preparation did not interfere with the detection of viable microorganisms.

**USP <1223> Container Closure Integrity Testing**

Positive control syringes were laser-drilled (5 ± 2 μm) to simulate realistic container defects; defect-free syringes served as negative controls. After 110 days, two syringes each of BD, Zero Residual, StaClear and ClearJect were immersed in 0.15 g/mL methylene blue (Sigma-Aldrich) and subjected to three vacuum/atmospheric cycles (650 ± 50 mbar, 10 min each) in a desiccator over one hour. After rinsing, internal contents were transferred to Eppendorf tubes. Aliquots (60 μL) from each sample, controls, and a blank (HPLC-grade water) were loaded into a 96-well plate, and absorbance at 664 nm was measured using a SpectraMax ABS Plus spectrophotometer. A standard curve of methylene blue concentrations was used to determine the concentration of methylene blue inside test samples. The limit of detection (LOD) was established using the ‘limit of the blank’ method; and containers were defined as intact if the observed absorbance was below the limit of detection.
